# Bacterial Multidrug Efflux Pumps: Much More Than Antibiotic Resistance Determinants

**DOI:** 10.3390/microorganisms4010014

**Published:** 2016-02-16

**Authors:** Paula Blanco, Sara Hernando-Amado, Jose Antonio Reales-Calderon, Fernando Corona, Felipe Lira, Manuel Alcalde-Rico, Alejandra Bernardini, Maria Blanca Sanchez, Jose Luis Martinez

**Affiliations:** Departamento de Biotecnología Microbiana, Centro Nacional de Biotecnología, CSIC, Darwin 3, Cantoblanco, 28049 Madrid, Spain; pblanco@cnb.csic.es (P.B.); shernando@cnb.csic.es (S.H.-A.); jareales@farm.ucm.es (J.A.R.-C.); fcorona@cnb.csic.es (F.C.); felipe.lira@cnb.csic.es (F.L.); malcalde@cnb.csic.es (M.A.-R.); abernardini@cnb.csic.es (A.B.); bsanchez@cnb.csic.es (M.B.S.)

**Keywords:** multidrug efflux pumps, MDR, quorum sensing, antibiotic resistance, solvent tolerance, bacteria/plant interactions

## Abstract

Bacterial multidrug efflux pumps are antibiotic resistance determinants present in all microorganisms. With few exceptions, they are chromosomally encoded and present a conserved organization both at the genetic and at the protein levels. In addition, most, if not all, strains of a given bacterial species present the same chromosomally-encoded efflux pumps. Altogether this indicates that multidrug efflux pumps are ancient elements encoded in bacterial genomes long before the recent use of antibiotics for human and animal therapy. In this regard, it is worth mentioning that efflux pumps can extrude a wide range of substrates that include, besides antibiotics, heavy metals, organic pollutants, plant-produced compounds, quorum sensing signals or bacterial metabolites, among others. In the current review, we present information on the different functions that multidrug efflux pumps may have for the bacterial behaviour in different habitats as well as on their regulation by specific signals. Since, in addition to their function in non-clinical ecosystems, multidrug efflux pumps contribute to intrinsic, acquired, and phenotypic resistance of bacterial pathogens, the review also presents information on the search for inhibitors of multidrug efflux pumps, which are currently under development, in the aim of increasing the susceptibility of bacterial pathogens to antibiotics.

## 1. Introduction

Resistance to antibiotics can be explained in biochemical terms as the inability of a given antibiotic to reach its microbial target at an adequate concentration for inhibiting the target's activity. Within this scope, there are two main ways of acquiring resistance: decreasing the affinity of the target for the antibiotic (mutations in genes encoding the antimicrobial targets) or diminishing the active concentration of the antibiotic inside the cell. For the latter, the mechanisms of resistance can be broadly classified in three categories: (i) production of hydrolytic or modifying enzymes; (ii) mutations in antibiotics' transporters impeding their entrance; and (iii) use of energy-dependent efflux pumps to extrude the antibiotics, impairing their accessibility to the target. Efflux pumps were firstly described as a mechanism of resistance to tetracycline in *Escherichia coli* [1]. However, nowadays it is well known that efflux pumps constitute the most ubiquitous type of resistance element, present in all organisms from bacteria to mammals, among those that have been described [2,3].

In several cases, the acquisition of resistance to multiple antimicrobials is the consequence of the presence in the same genetic mobile element of several genes, each one encoding a different resistance determinant (co-resistance). However, in some occasions the same determinant can confer resistance to different antimicrobials (cross-resistance). The most conspicuous examples of determinants conferring cross-resistance to different antibiotics are multidrug resistance (MDR) efflux pumps. As stated above, these transporters are present in all organisms, including, in addition to bacterial pathogens [4,5], human cells [6] and eukaryotic pathogens such as *Candida albicans* [7] or *Plasmodium falciparium* [8]. It is to be noticed that the efflux systems can actively extrude a variety of compounds; not just conventional antimicrobials, but also non-antibiotic substrates such as dyes, detergents, heavy metals, and organic solvents, among others [9,10,11].

**Figure 1:**
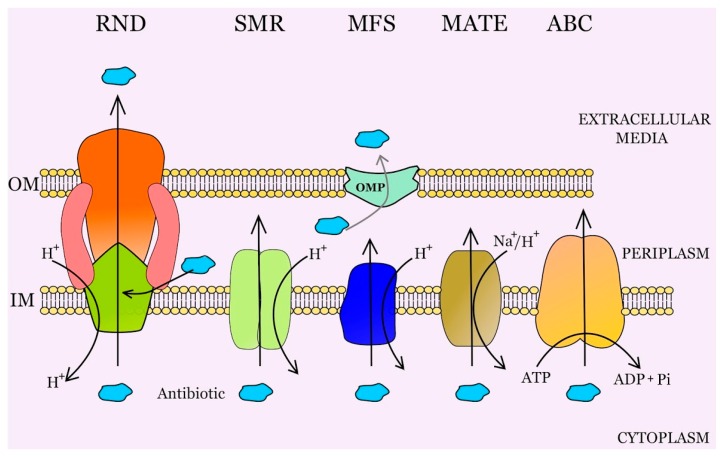
Schematic representation of the main types of bacterial efflux systems. Schematic illustration of the five major families of efflux transporters: the resistance- nodulation-division (RND) family, the small multidrug resistance (SMR) family, the major facilitator superfamily (MFS), the multidrug and toxic compound extrusion (MATE) family and the adenosine triphosphate (ATP)-binding cassette (ABC) superfamily. IM: Inner membrane. OM: Outer membrane. OMP: Outer membrane protein.

In the prokaryotic kingdom there are five major families of efflux transporters (Figure 1): the adenosine triphosphate (ATP)-binding cassette (ABC) superfamily [12], the resistance-nodulation-division (RND) family [13], the small multidrug resistance (SMR) family [14], the major facilitator superfamily (MFS) [15], and the multidrug and toxic compound extrusion (MATE) family [16]. These families have been defined on the basis of their sequence similarity, substrate specificity, number of components (single or multiple), number of transmembrane-spanning regions, and energy source. The ABC family utilizes ATP hydrolysis to drive the export of substrates, whereas the other families utilize the proton motive force as the energy source. The MFS, ABC, SMR, and MATE families are widely distributed in Gram-positive and Gram-negative bacteria, while the RND superfamily is specific to Gram-negative microorganisms. The members of the RND family are always forming part of a tripartite complex spanning across the two membranes of Gram-negative bacteria [17]. In Gram-positive bacteria, the MFS family is the most relevant efflux pump group, the best studied members of this family being NorA from *Staphylococcus aureus* and PmrA from *Streptococcus pneumoniae* [18,19,20].

It is important to remark that efflux pumps are ancient, highly-conserved determinants, which have been selected long before the recent use of antibiotics for the therapy of human infections. These characteristics suggest that the role of efflux pumps as relevant antibiotic resistance determinants in bacterial pathogens is a recent event, likely secondary to other functional roles with relevance to bacterial physiology [3,21,22]. Some of these functional roles not directly linked to antibiotic resistance are discussed below.

## 2. Multidrug Efflux Pumps and Antibiotic Resistance

The possibility that bacteria can acquire resistance by extruding antibiotics was firstly described in 1980, when McMurry and colleagues described the existence of plasmid-encoded proteins capable of extruding tetracycline and conferring resistance to this antibiotic in *E. coli* [1]. Although the mechanism was novel, its finding still fitted into the paradigm of acquisition of resistance genes: they confer resistance to one structural family of antibiotics and are acquired through horizontal gene transfer (HGT), likely from antibiotic producers [23,24]. Nevertheless, the finding two years later of a chromosomally-encoded efflux pump, not acquired through HGT and conferring resistance to several drugs [25], challenged this paradigm. Indeed, differing to classical resistance elements, multidrug efflux pumps are present in all organisms and are well conserved among the different members of a given species.

**Figure 2:**
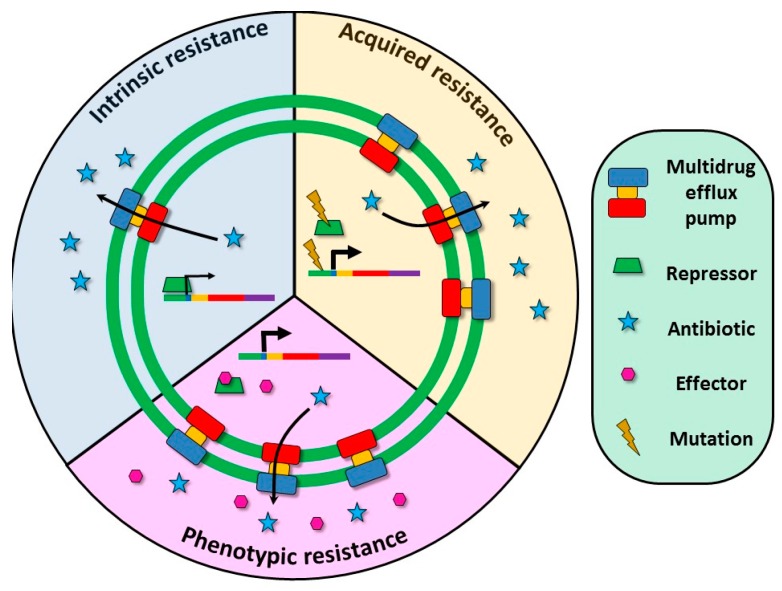
Role of multidrug efflux pumps in antibiotic resistance. Expression of efflux pumps is frequently down-regulated by transcriptional repressors encoded upstream of the pump operon. Consequently, MDR efflux pumps can contribute to the phenotype of antibiotic resistance at different levels, depending on their expression level. Intrinsic resistance: Some MDR efflux pumps, such as *P. aeruginosa* MexAB-OprN [62] or *E. coli* AcrAB-TolC [63], present a basal level of expression, enough for contributing to the intrinsic antimicrobial resistance of these microorganisms (blue in the Figure). Acquired resistance: De-repression of the expression of the efflux pumps can be achieved by mutations at the regulatory proteins, rendering stable acquired resistance (yellow in the Figure). Phenotypic resistance: The expression of efflux pumps can by triggered in the presence of specific inducers, rendering transient phenotypic resistance (pink in the Figure).

Expression of MDR efflux pumps is tightly regulated [26]; some of them are expressed at moderate levels, in which case they contribute to intrinsic resistance, whereas for some others the level of expression is very low, at least under laboratory growing conditions. In both cases, a higher level of expression and hence of antimicrobial resistance can be achieved in two ways (Figure 2); transiently, in the presence of inducers of the efflux pumps' expression (see below), or constitutively, due to mutations in the elements that downregulate their expression [27]. Several studies have highlighted the contribution of MDR efflux pumps to the acquisition of multidrug resistance by different pathogens [5,28,29,30]. In this regard, it is important to note that while HGT is an important event in spreading resistance, this mechanism requires the presence of a donor of the resistance gene and is less suitable to occur during single-pathogen infections, including chronic infections. For this type of infections, mutation-driven resistance is likely a more frequent mechanism to acquire resistance to antibiotics. Indeed, during antibiotic treatments of infected and/or colonized patients, bacteria may develop resistance to multiple drugs [31]. This observation can be explained either by the *in vivo* acquisition of genetic mobile elements carrying multiple antibiotic resistance genes [32], or by the selection of resistance mutations conferring a multi-resistance phenotype. Indeed, some recent studies associate the overexpression of MDR efflux pumps with the increasing clinical cases of MDR bacterial infections [33,34,35]. Although expression of a single efflux pump can confer resistance to multiple antimicrobials, simultaneous overexpression of more than one efflux system has been described in *Pseudomonas aeruginosa* [36,37] and in *Stenotrophomonas maltophilia* clinical isolates [38].

Due to their role in antibiotic resistance, efflux pumps can be considered as potentially effective antibacterial targets, and the development of bacterial efflux pump inhibitors may help to improve the therapeutic armamentarium against resistant microorganisms [39,40,41,42]. However, efflux pumps differ from other mechanisms of resistance (such as beta-lactamases) that operate over a specific family of antibiotics; any single efflux pump can extrude a wide range of different families of antibiotics, so that its inhibition will increase the bacterial susceptibility to several antimicrobials [43,44].

There exist different possibilities for inhibiting the activity of MDR determinants [27]. One could be the inhibition of the energy sources required for the activity of efflux pumps: the membrane potential and the generation of ATP. However, potential inhibitors of these targets will be toxic as well for human cells and consequently are clinically useless [44]. The search for bacteria-specific elements coupling the energized state of the inner membrane to the activity of efflux pumps as TonB in *P. aeruginosa* may help in finding valuable targets [45].

Another possibility to inhibit efflux pumps’ activity is by developing compounds able to compete with the antibiotics for their extrusion. One of the first members of this type of inhibitors is the dipeptide amide phenylalanine-arginine-β-naphthylamide (PAβN), which inhibits several [46,47], but not all [48], RND efflux pumps. This synthetic molecule is a competitive inhibitor that binds to the same site used by the efflux pump to bind the antibiotic it extrudes. However, this molecule and its derivatives are too toxic to be used in therapy [39]. Other molecules with efflux pump inhibitory activity are the pyridopyrimidines and arylpiperazines, and there have been efforts to optimize them for therapeutic use [49,50,51]. Differing from the previously described dipeptide amides, which just impede the action of a subset of antibiotics among those extruded by the efflux pumps (the ones binding at the same site of the efflux pump), pyridopyrimidines increase the susceptibility to all substrates of such efflux pumps, indicating a different mechanism of action [39]. As we will see below, plant-produced compounds are substrates and inducers of efflux pumps [52,53]. Moreover, it has been shown that plant extracts contain a variety of efflux pump inhibitors [54,55]. Indeed, *in silico* screening of plant compounds structurally similar to PAβN allowed the identification of plumbagin nordihydroguaretic acid and, to a lesser degree, shikonin as potentially useful inhibitors of MDR efflux pumps [56]. Although none of these efflux pumps inhibitors are still available, their use may be useful for increasing the susceptibility of different bacterial species to antibiotics [57,58].

Another way to avoid the extrusion of antibiotics by efflux pumps is to modify the antibiotic molecule itself in order to reduce its affinity for the efflux pumps. In the tetracycline and macrolide families, the new compounds of the glycylcycline and ketolide classes differ from their progenitors in having lower affinities for specific efflux pumps [59]. Tigecycline is not extruded at a high level by MFS efflux pumps of both Gram-negatives and Gram-positives [60], while telithromycin has significantly increased activity against bacterial species presenting elevated macrolide efflux [61]. Efforts in the search of efflux pumps inhibitors may help in reducing the impact of MDR efflux pumps in the acquisition of antibiotic resistance by bacterial pathogens.

## 3. Multidrug Efflux Pumps Are Not Just Antibiotic Resistance Elements

Although most studies on multidrug efflux pumps focus on their role as antibiotic resistance elements, they can confer resistance to other compounds. The best studied is resistance to heavy metals, which have been reviewed in detail [11] and will not be discussed here. Another biological process in which efflux pumps have a relevant role is the biodegradation of organic pollutants by protecting the bacterial cell from toxic components of such pollutants. One of the best studied organisms with biodegradative potential is *Pseudomonas putida* DOT-TE1, a strain able to resist high concentrations of solvents, partly because of the activity of TtgABC, a tripartite RND efflux pump implicated in toluene tolerance in this strain. Expression of TtgABC is down-regulated by the TtgR transcriptional repressor; mutations inactivating TtgR lead to overexpression of the efflux pump, making *P. putida* DOT-TE1 more resistant than the wild-type strain to chloramphenicol, nalidixic acid, and tetracycline [64]. Further, TtgABC expression is induced by erythromycin, colistin, ceftazidime, and ciprofloxacin, as well by narigenin and toluene [65]. Altogether, these data indicate the multiple roles that this efflux pump may have in different habitats, each one presenting specific inducers of its expression. Another example of efflux pumps involved in biodegradation is NepAB from *Arthronobacter nicotinovorans.* This efflux pump, which belongs to the SMR family, extrudes methylamine, the last compound resulting from the biodegradation of nicotine [66].

Even in the case of pathogens, efflux pumps can extrude compounds that are not regularly present inside the human host. For instance, it has been shown that the selection of mutants of *P. aeruginosa* that resist organic solvents leads to overexpression of antibiotic efflux pumps [67]. Taking into account that *P. aeruginosa* strains exhibit pathogenic and biodegradative properties independently of their origin, it is important to notice that organic solvents and contaminants can co-select resistant strains through overexpression of efflux pumps in non-clinical environments [68], highlighting the potential role that these ecosystems may have in the selection of antibiotic resistance.

It is generally assumed that multidrug efflux pumps extrude toxic compounds that are coming from outside the bacteria (antimicrobials produced by competitors, heavy metals, and organic pollutants, among others). However, they can also extrude, and its expression can be induced by, endogenous metabolites. The expression of the efflux pump AcrAB is induced by 2,3-dyhidroxibenzoate, an intermediary metabolite of the synthesis of the siderophore enterobactin [69]. In line with this induction, it has been shown that AcrB, AcrD, and MdtABC are involved in the extrusion of enterobactin [70], and this siderophore is accumulated in a TolC-defective mutant [71]. Similarly, the efflux pump MexGHI is induced by pyocyanine, a secondary metabolite of *P. aeruginosa* [72] and is involved in the detoxification of anthranilate [73], a precursor of PQS (*Pseudomonas* Quinolone Signal), a quorum sensing molecule of *P. aeruginosa*. Another efflux pump of *P. aeruginosa*, MexEF-OprN, extrudes kynurenine, an intermediate in the pathway of tryptophan degradation and a precursor of anthranilate [74]. Altogether these results point to a role of efflux pumps in the detoxification of damaging endogenous metabolic intermediates.

In addition to extruding toxic compounds, different works have shown that efflux pumps can be mediators in cell-to-cell communication processes through the extrusion of signalling molecules. This situation has been studied in more detail in the case of *P. aeruginosa*, a bacterial species that presents two interlinked quorum sensing systems. In one of them, the signal molecules are homoserine lactones with different acyl chain modifications (AHLs), whereas in the other, the signal is the previously mentioned PQS. It has been reported that MexAB-OprM extrudes 3-oxo-C_12_-HSL, an AHL signal molecule with a large side acyl chain. Since the quorum sensing response is relevant for *P. aeruginosa* virulence, resistant mutants overexpressing MexAB, which accumulate lower quantities of this quorum sensing signal, are less virulent [75,76,77]. Similarly, it has been shown that the deletion of MexGHI reduces the production and secretion of AHLs [73], although a direct role of the efflux pump in the extrusion of these signals has not been demonstrated. As stated above, this efflux pump can extrude anthranilate, a PQS precursor, linking in this way the two quorum sensing regulation pathways of *P. aeruginosa* [78]. It is to be noted that this effect on PQS secretion is not specific for MexGHI, since it has been demonstrated that MexEF-OprN can extrude kynurenine, another PQS precursor (see above).

Altogether, this highlights the great versatility of efflux pumps in extruding a large variety of compounds, a relevant feature for the bacterial adaptation to an assorted range of habitats.

## 4. The Role of Efflux Pumps on Biocide Resistance

Biocides are a group of antimicrobials used for disinfection, antiseptic, and preservative purposes. Although some of them, such as chlorhexidine, have been used in skin decontamination for preventing infections [79,80,81], biocides are not used for treating *in host* infections. Because of this, the regulations for the utilization of these compounds are not as strict as in the case of antibiotics, and they are widely used without major restrictions in the food industry, veterinary, household-cleaning compounds, or hand and teeth washing, among other applications. One aspect that is a matter of concern is the possibility that the presence of biocides may select antibiotic-resistant microorganisms [82,83,84,85]. Biocides usually present several targets and in occasions interact directly with the cell envelopes. Consequently, resistance should be difficult to achieve. On the other hand, classical definitions of antibiotic resistance based on breakpoints are not available in the case of biocides [86] and appropriate definitions of biocide resistance require the study of a large number of isolates [87,88]. Since biocides present multiple targets [89], each biocide resembles itself as a combination of different antimicrobials, and for this type of combination, cross-resistance can be achieved by overexpression of MDR efflux pumps. It is then conceivable that efflux pumps might have a role in the resistance to biocides. Indeed, expression of efflux pumps was shown to decrease the efficiency of distinct classes of biocides, including chlorhexidine digluconate, hydrogen peroxide, benzalkonium chloride, chloroxylenol, iodine compounds, triclosan, quaternary ammonium compounds, phenolic parabens, and DNA intercalating agents [83,90,91,92]. This mechanism of resistance to biocides mediated by their extrusion is not restricted to a specific group of bacteria. Efflux pump-mediated biocide resistance has been described in a large range of environmental and clinically relevant bacteria. Among the best studied systems for extruding biocides, we can highlight MexAB-OprM, MexCD-OprJ, and MexEF-OprN from *P. aeruginosa* [93,94,95], AcrAB-TolC, AcrEF-TolC, and EmrE from *Escherichia coli* [96,97], SmeDEF from *Stenotrophomonas maltophilia* [98], or NorA and MepA from *Staphylococcus aureus* [99]. It is important to note that several reports have shown that bacteria can make use of the same efflux pumps for extruding antibiotics and biocides and that biocides can select antibiotic-resistant mutants that overexpress such efflux pumps [83,85,100,101]. The intensive use of these compounds, together with the stability of the biocides in natural ecosystems, may promote the emergence of resistant organisms—not just to the biocides, but also to antibiotics [102].

In addition to selecting antibiotic-resistant mutants, some works have shown that biocides can also induce the expression of MDR efflux pumps, which on occasion renders a phenotype of transient antibiotic resistance. For instance, it has been shown that pentachlorophenol and triclosan can induce the expression the *P. aeruginosa* efflux pump *mexAB-OprM* through their binding to NalC, one of the regulators of the expression of this efflux pump [103]. The exposure to chlorinated phenols and chlorinated phenol-based disinfectants results in the acquisition of a phenotype of transient antibiotic resistance in *P. aeruginosa*. This increased antibiotic resistance, shown when *P. aeruginosa* is exposed to chlorinated phenols, might be relevant for the survival of *P. aeruginosa* in places such as health care units, where combinations of chlorophenols and antibiotics are used [104].

The expression of SmeDEF, the most important MDR efflux pump known to confer antibiotic resistance in *S. maltophilia* [105], is also induced by biocides. The binding of triclosan, a known substrate of this efflux pump [98] to its transcriptional repressor SmeT [106], causes the overexpression of the SmeDEF efflux pump and reduces *S. maltophilia* susceptibility to quinolones [107]. Benzalkonium chloride, another common biocide, triggers the expression of *smeDEF*. However, it does not produce any relevant change in the susceptibility of *S. maltophilia* to antibiotics. This is likely due to the fact that the concentrations of the biocide required for such an effect are in the range of the lethal concentration for benzalkonium [108].

## 5. The Functional Role of Multidrug Efflux Pumps in Non-Clinical Environments: Bacteria-Plant Interactions

The presence of MDR efflux pumps in bacteria is not restricted to clinical environments, which are characterized by the presence of high levels of antibiotics. Even more, the bacterial species presenting larger numbers of genes encoding efflux pumps are inhabitants of natural environments, such as plants or soil. Nevertheless, there is no correlation between the number of MDR efflux pumps found in each genome and the antibiotic resistance phenotype observed [109,110]. This finding suggests that, besides conferring resistance to antibiotics, efflux pumps may have functions relevant to bacterial behaviour in natural environments. Even in the case of opportunistic pathogens with an environmental origin, the activity of efflux pumps may be relevant for bacterial physiology in natural (non-clinical) ecosystems. For instance, it has been described that the SmeDEF efflux pump, the most important quinolone resistance determinant of *S. maltophilia*, is involved in bacteria/plant interactions, since a mutant lacking *smeE* is unable to colonize the roots of the plants [53]. Further supporting the role of efflux pumps in bacteria/plant interactions is the finding that flavonoids can induce SmeDEF expression by binding to its transcriptional repressor SmeT [53]. This feature is not restricted to a given organism—the flavonoid-responsive RND family of efflux pumps includes several members, such as AcrAB from *Erwinia amylovora*, IfeAB from *Agrobacterium tumefaciens*, MexAB-OprM from *Pseudomonas syringae*, BjG30 from *Bradyrhizobium japonicum,* and EmrAB in *Sinorhizobium meliloti* [111,112,113,114,115], among others. Further supporting the role of this efflux pump in bacteria/plant interactions, it has been reported that *E. amylovora*, an enterobacterium that causes fire blight on species of the Rosaceae family, has an AcrAB efflux pump, which confer resistance to phytoalexins, and that is required for successful colonization of the plants and for bacterial virulence [116,117]. This finding is in agreement with the idea that the ability to deal with toxic compounds is one of the key traits for survival in the rhizosphere, and efflux pumps may have a relevant role for achieving resistance to these toxic compounds. To note here that the bacterial response to environmental injuries might be complex, with several elements involved. For instance, *E. amylovora*, besides AcrAB, presents two other efflux pumps, MdtABC and MdtUVW, which are induced by the polyphenol tannin during their growth *in planta.* It has been shown that *mdtABC*- and *mdtUVW*-deficient mutants have a reduced ability to multiply in apple rootstock, suggesting their implication in resistance to plant antimicrobial compounds [118].

Other examples of efflux pumps involved in bacteria/plant interactions are highlighted below. In *A. tumefaciens*, the IfeAB efflux pump is involved in the competitive colonization of alfalfa roots and can confer measurable ecological benefits to these bacteria in an environment where flavonoids are present [114]. The EmrAB efflux system in *S. meliloti* is induced by flavonoids and bacterial symbiosis with *Medicago sativa* is impaired when *emrR,* the gene encoding the TetR repressor of this efflux pump, is deleted [115,119]. Another multidrug efflux system of *S. meliloti* that plays an important role in nodulation competitiveness by mediating resistance toward antimicrobial compounds produced by the host plant is SmeAB [120]. The BjG30 efflux pump from *B. japonicum* may play a role in the early stage of symbiosis of this microorganism with soybean by balancing the dual functions of genistein as both a *nod* gene inducer and as a toxic compound [112]. Notably, *B. japonicum* presents another efflux pump, BdeAB, which seems to be involved in the symbiotic nitrogen-fixation activity of this microorganism in soybean; mutants deficient in this efflux pump, in addition of presenting symbiotic defects, are more susceptible to aminoglycosides [121], showing that antibiotic resistance is interlinked with other relevant functions of efflux pumps. *Erwinia chrysanthemi* is another example of the need of efflux pumps to colonize plant tissues. The infection by *E. chrysanthemi* causes salicylic acid accumulation in the host, leading to an amplification of the plant defence response and the production of pathogenesis-related proteins and toxic antimicrobial compounds. The combination of salicylic acid and its precursors activates the expression of multidrug efflux pump-encoding genes and enhances the survival of the bacterium [122].

It is important to highlight that efflux pumps may play a double functional role by modulating bacteria/plant and intermicrobial interactions. Indeed, a *tolC* mutant of *E. chrysanthemi* is defective in the efflux of berberine, an antimicrobial plant compound, and it is unable to cause plant tissue maceration *in planta*. In addition, this mutant is impaired for competing with the microbial community present in the same ecosystems, indicating that these efflux pumps have a role in microbial interspecific competition [123]. In line with the potential role of efflux pumps on bacterial competition, it has been shown that an *E. chrysanthemi* mutant defective in the ABC transporter YbiT conserves virulence in potato tubers but is less infectious than the wild type strain when growing together with saprophytic bacteria such as *P. fluorescens* or *P. putida*, possibly because this efflux pump can extrude toxic compounds produced by these bacteria [124].

Altogether, these results indicate that bacterial efflux pumps, in addition to being antibiotic resistance determinants, are relevant elements for the physiology of microorganisms in natural ecosystems, in the cases above described in bacteria/plant interactions.

## 6. Induction of the Expression of Efflux Pumps

**Figure 3:**
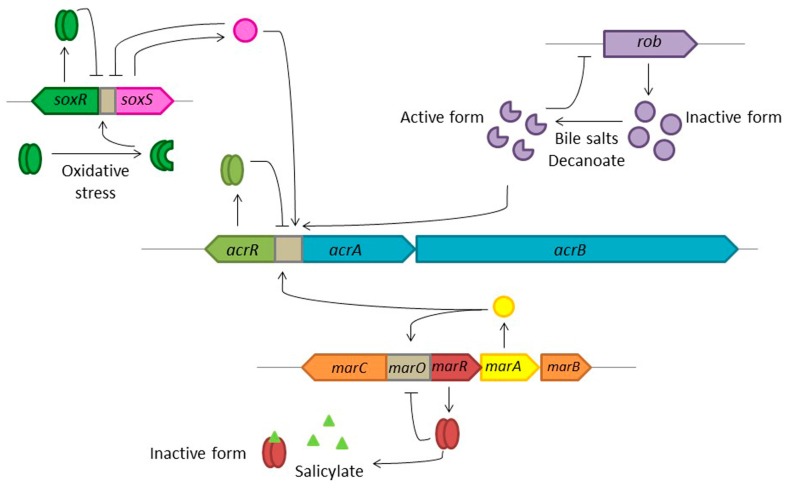
Schematic representation of the regulation of the expression of the *E. coli acrAB* multidrug efflux pump. AcrAB-TolC is a tripartite complex formed by AcrA, a membrane fusion protein, AcrB, a cytoplasmic membrane protein, and TolC, an outer membrane protein. *acrA* and *acrB* are part of the same operon, which is negatively regulated by the dimeric protein AcrR. The global regulators MarA, SoxS, and Rob can activate *acrAB* expression in response to different environmental signals. MarA is encoded by the second gene of the *marRAB* operon, the expression of which is repressed by MarR through its binding to its operator *marO*. The presence of salicylate inactivates MarR, leading to the expression of *marRAB*. MarA increases its own transcription and activates the expression of *acrAB*. SoxR and SoxS constitute an oxidative response system [136]. In the absence of signals, the homodimer SoxR represses *soxS* expression, but under oxidative stress conditions, SoxR is oxidized and becomes an activator of *soxS* transcription [137]. SoxS binds to the *acrAB* promoter region and induces its expression. Rob is constitutively expressed but remains in an inactive form, unless an effector such as decanoate or bile salts is present. Those effectors bind and produce conformational changes that activate Rob, inducing *acrAB* expression.

Expression of efflux pumps is usually tightly down-regulated, which means that transient high-level expression is achieved just in the presence of the right effectors. Knowing these effectors may be useful to infer the biological roles that these efflux pumps may have, besides resistance to antibiotics. We have already discussed induction of the expression of efflux pumps by plant-produced compounds and by biocides (see above). In addition, it is important to notice that among those effectors that trigger expression of MDR efflux pumps, some might be relevant during infections [26], which links resistance with the virulence of bacterial pathogens [125]. Among them, the RND transporter AcrAB in *E. coli* has been extensively studied as a prototype of the family, which exports dyes, detergents (including bile salts), chloramphenicol, tetracyclines, macrolides, β-lactams, fluoroquinolones, and organic solvents [126]. Its expression is negatively regulated by AcrR [127], and positively regulated by three XylS/AraC family regulators [128], MarA, SoxS, and Rob (Figure 3). Chemicals commonly found in the intestinal tract, like decanoate or bile salts, are able to induce the expression of *acrAB* by binding and producing conformational alterations that render the post-translational activation of Rob [128]. Expression of *acrAB* is also induced during situations in which the cell is under oxidative stress. This induction is SoxRS dependent. Oxidation of the iron–sulfur clusters in SoxR by superoxide species produces the induction of SoxS expression, which binds to the *acrAB* promoter and induces its expression [129]. The induction of efflux pumps, and consequently antibiotic resistance, in the presence of bile, cationic peptides, or fatty acids [130,131,132], is clinically relevant because bacteria can encounter these compounds inside the host and therefore might display a phenotype of transient resistance in the course of an infection. Notably, expression of *acrAB* is triggered through the MarA regulator by salicylate, a phenolic phytohormone implicated in plant growth/development/defence against pathogens, which presents anti-inflammatory properties. Binding of salicylate to MarR, a local repressor of the *marRAB* operon, causes conformational changes in the protein which leads to disassociation of MarR from the *marRAB* promoter. As a consequence, expression of *marA* is de-repressed, which activates expression of *acrAB* [133].

Phylogenetically close to *E. coli*, the enterobacterial pathogen *Salmonella enterica* serovar Typhimurium presents at least nine multidrug efflux pumps. Among these pumps, AcrAB, the ortholog of the *E. coli* efflux pump with the same name, contributes to antimicrobial resistance and has a wide substrate spectrum that includes antibiotics, dyes, and detergents. AcrAB in *Salmonella* is induced by indole, bile, and *E. coli* metabolites [131]. The bile-mediated induction of *acrAB* expression is dependent on RamA, a regulator belonging to the AraC/XylS family that activates the expression of *acrAB* and *tolC* by directly binding their promoter regions. The transcription of *ramA* is itself repressed by RamR, which is encoded by the gene located immediately upstream of *ramA*. Both bile and indole trigger *ramA* transcription [131,134]. It is to be noticed that both bile and indole are present in the gut; bile salts are produced by the host and indole is secreted by many enteric bacterial species, being detected in human faeces. Therefore, RamR may be required by *Salmonella* to detect environmental cues and for subsequent induction of the AcrAB-TolC system, resulting in bacterial adaptation for growing in the intestine [135]. In addition to RamA, AcrAB expression is regulated by three activators, MarA, SoxS, Rob, and one repressor, AcrR [127]. This complex regulation may help *Salmonella* for a fine-tuning response to environmental signals and thus to adapt itself to different environments [135]. For instance, paraquat induces *acrAB* expression via SoxS, not affecting *ramA*. SoxS is proposed to bind to the upstream region of *acrA* and to directly induce *acrAB*. This suggests that RamA and SoxS competitively bind to the upstream region of *acrA*.

Another important gut pathogen is *Campylobacter jejuni*. Among the known antibiotic resistance mechanisms of this microorganism, the CmeABC efflux pump is a relevant player and confers resistance to structurally-diverse antibiotics and toxic compounds [138], including those naturally present in its animal host, as bile salts [130]. CmeABC belongs to the RND family of efflux transporters and its expression is regulated by the transcriptional repressor CmeR, which binds to a specific site in the promoter region of *cmeABC* [139]. As it happens in the case of *acrAB*, bile salts, which are natural substrates of this efflux pump, are able to induce expression of *cmeABC*, promoting the dissociation of CmeR from its operator and allowing the transcription of the operon [139]. Induction by cholate, an unconjugated bile salt, is fully CmeR-dependent; however, induction by taurocholate, a conjugated bile salt, is not attributable to the release of CmeR-mediated repression, suggesting a CmeR-independent pathway. Induction of this efflux pump by bile salts confers resistance to diverse antibiotics, including cefotaxime, novobiocin, ciprofloxacin, and erythromycin. As in the case of *Salmonella* (see above), the presence of bile salts in the gut may decrease the susceptibility of *C. jejuni* to antibiotics *in vivo*. Like in the case of *acrAB*, salicylate also induces the expression of *cmeABC* in *C. jejuni* and promotes the emergence of quinolone-resistant mutants in this bacterial species [140]. This induction can be explained by the fact that salicylate inhibits the binding of CmeR to its operator DNA, although this inhibition is weaker than in the presence of bile salts.

Free-living bacteria, including opportunistic pathogens with an environmental origin, should respond to different signals and this may impact their behaviour in clinical and non-clinical ecosystems. For instance, *Pseudomonas aeruginosa* express several RND-type efflux systems, among which four, MexAB-OprM, MexCD-OprJ, MexEF-OprN, and MexXY-OprM are reported to be significant determinants of multidrug resistance [141]. MexEF-OprN expression is regulated by the transcriptional activator MexT [142]. Expression of this efflux pump is induced, via MexT, by nitrosative stress as well as by chloramphenicol, a nitrosated aromatic compound that probably mimics a nitrosated product of nitrosative stress [143]. Airway epithelial cells are known to produce NO upon stimulation by bacteria and it has been shown that *mexEF-oprN* is overexpressed when *P. aeruginosa* grows on human epithelial cells [144]. A distinctive feature of the *P. aeruginosa* MexXY-OprM system is that the expression of *mexXY* is induced by exposure to several of the antibiotics that target the ribosome and that this efflux system exports [145]. However, these antimicrobials do not interact with MexZ, the *mexXY* repressor [146]; expression of the efflux pump being induced just when the ribosome is functionally impaired [147].

The fact that the expression of MDR efflux pumps is induced by host-produced compounds suggests that they can play a role in the virulence of bacterial pathogens, a possibility that was discussed a decade ago [148]. Indeed, it has been shown that the *Vibrio cholerae* efflux pump VexB is the primary efflux system responsible for resistance to bile salts in this microorganism [149]. Since bile salts are present at the human gut, the activity of this efflux pump is a pre-requisite for *V. cholerae* infection. A similar situation happens with AcrAB, the main pump responsible for bile salts resistance in *Enterobacteriaceae* [150], which is required for the pathogenesis of *Salmonella enterica* serovar Typhimurium [151]. Notably this efflux pump is involved as well in the bacterial capability for forming biofilms [152,153]. A protective role to host antibacterial compounds has also been described in the case of *Neisseria gonorrhoeae*. In this organism, the MtrCDE efflux pump contributes to resistance to vertebrate antibacterial peptides [132,154], and FarAB is involved in resistance to long-chain fatty acids [155]. The activity of these efflux pumps contributes to the pathogenesis of *N. gonorrhoeae* [154,156]. Similarly, the *Campylobacter jejuni* CmeABC efflux pump confers resistance to bile salts, fatty acids, and detergents, and is needed for the colonization of the intestinal tract [157].

In addition to their protective role against host antibacterial compounds, efflux pumps may be involved in other aspects of bacterial virulence. The contribution of the MDR efflux pumps MexAB-OprM, MexCD-OprJ, MexEF-OprM, and MexXY to *P. aeruginosa* virulence has been studied by using knock-out mutants of each of them [158]. With the exception of *mexCD-OprJ*, all knock-out mutants were impaired in their capability of invading MDCK cells, the effect being higher in the case of the *mexAB-OprM* mutant, this efflux pump being essential for inducing lethal septicaemia in a murine model.

Together with their role in modulating the quorum-sensing response, and consequently bacterial virulence [74,75,77,159,160], these results support the notion that MDR efflux pumps, besides contributing to the resistance of bacterial pathogens, are major contributors to their pathogenicity [148]. Inhibition of efflux pumps may then allow to both increase the susceptibility to antibiotics and reduce the virulence of bacterial pathogens.

As discussed above, expression of efflux pumps can be triggered by a variety of effectors, and even physiological situations as it happens in the case of nitrosative stress or ribosome stalling. Finding these effectors may help to elucidate the regulatory mechanisms that control the expression of efflux pumps in the absence of antibiotics. It may allow predicting situations of transient antibiotic resistance, when the inducers are present [161].

## 7. Conclusions

Multidrug efflux pumps are ancient elements encoded in the chromosomes of microorganisms. They can confer resistance to antibiotics at different levels: intrinsic resistance, acquired resistance, and transient induced phenotypic resistance. In addition, multidrug efflux pumps display different functions with relevance to bacterial adaptation to different habitats. Some of these functions, such as resistance to heavy metals, biocides, or solvents, resemble antibiotic resistance, since they are adaptive responses to different types of external injuries, whereas others are related to internal detoxification of intermediate toxic bacterial metabolites. In addition, some efflux pumps are involved in antimicrobial or even interkingdom signalling. Among the latter, it is important to mention that different efflux pumps are involved in bacterial virulence, in both plant and animal hosts. Altogether, currently available information supports the notion that, besides contributing to antibiotic resistance, multidrug efflux pumps display a variety of functions with relevance to bacterial behaviour in different ecosystems.
